# Teaching NeuroImages: Absence of bilateral internal carotid arteries with multiple aneurysms

**DOI:** 10.1002/ccr3.6882

**Published:** 2023-01-23

**Authors:** Yang Gao, Jianhua Yuan, Jian‐Zhi Wang

**Affiliations:** ^1^ Department of Pathophysiology, School of Basic Medicine, Key Laboratory of Education Ministry of China/Hubei Province for Neurological Disorders Tongji Medical College, Huazhong University of Science and Technology Wuhan China; ^2^ Department of Radiology Guangdong Integrated Traditional Chinese and Western Medicine Hospital Foshan China; ^3^ Co‐innovation Center of Neuroregeneration Nantong University Nantong China

## Abstract

Absence of bilateral internal carotid arteries is a rare congenital vascular dysplasia. We reported the case and present some typical and exquisite neuroimges for teaching.

A 74‐year‐old male patient presented with aggravated weakness of both lower limbs for days. He was suspected diagnosis of cerebral ischemic disease and craniocervical CT angiography (CTA) were conducted. CTA revealed the absence of bilateral internal carotid arteries (ICAs) with compensated artery dilatation, multiple aneurysms, and sclerosis in cephalic and cervical arteries, without acute infarcts in diffusion‐weighted images (DWI) (Figure 1). CT scan also revealed hypoplastic carotid canals at both sides in bone window (Figure 2). With the informed risk of aneurysm rupture, the patient insisted on being discharge after the limbs weakness was improved by symptomatic treatments for a week. Since then, no stroke happened during a year of follow‐up.

**FIGURE 1:**
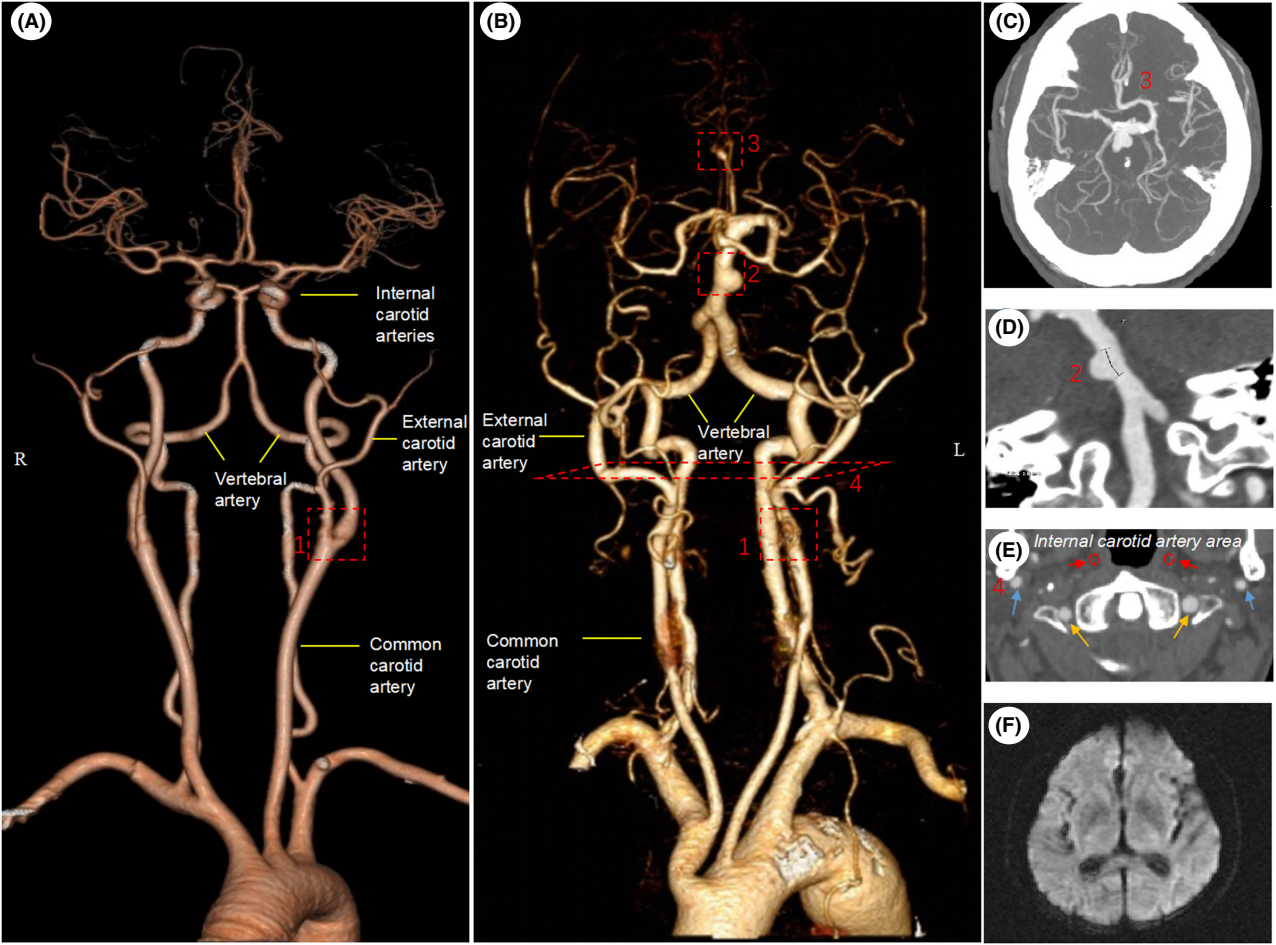
Absence of bilateral internal carotid arteries with multiple intracranial aneurysm but no acute infarction. (A) Three‐dimensional CTA assessment shown physiological anatomy of six large vessels supplying the brain, in which the common carotid artery is further divided into external carotid arteries (ECA) and ICA. (B) The patient had no ICA (Area 1) with enlarged vertebrobasilar arteries and ECA. Aneurysm appeared in basilar artery (Area 2) and left anterior cerebral artery A2 segment (Area 3). (C) Intracranial vascular reconstructed with maximum intensity projection (MIP) (D) Curvature plane reconstruction for basilar artery aneurysm (E) Axial location of bilateral ICA running area (red arrow, no imaging), external carotid artery (blue arrow) and vertebral artery (yellow arrow) as in panel B‐Area 4 dotted. (F) Diffusion weighted imaging (DWI) showed no acute cerebral infarction.

**FIGURE 2:**
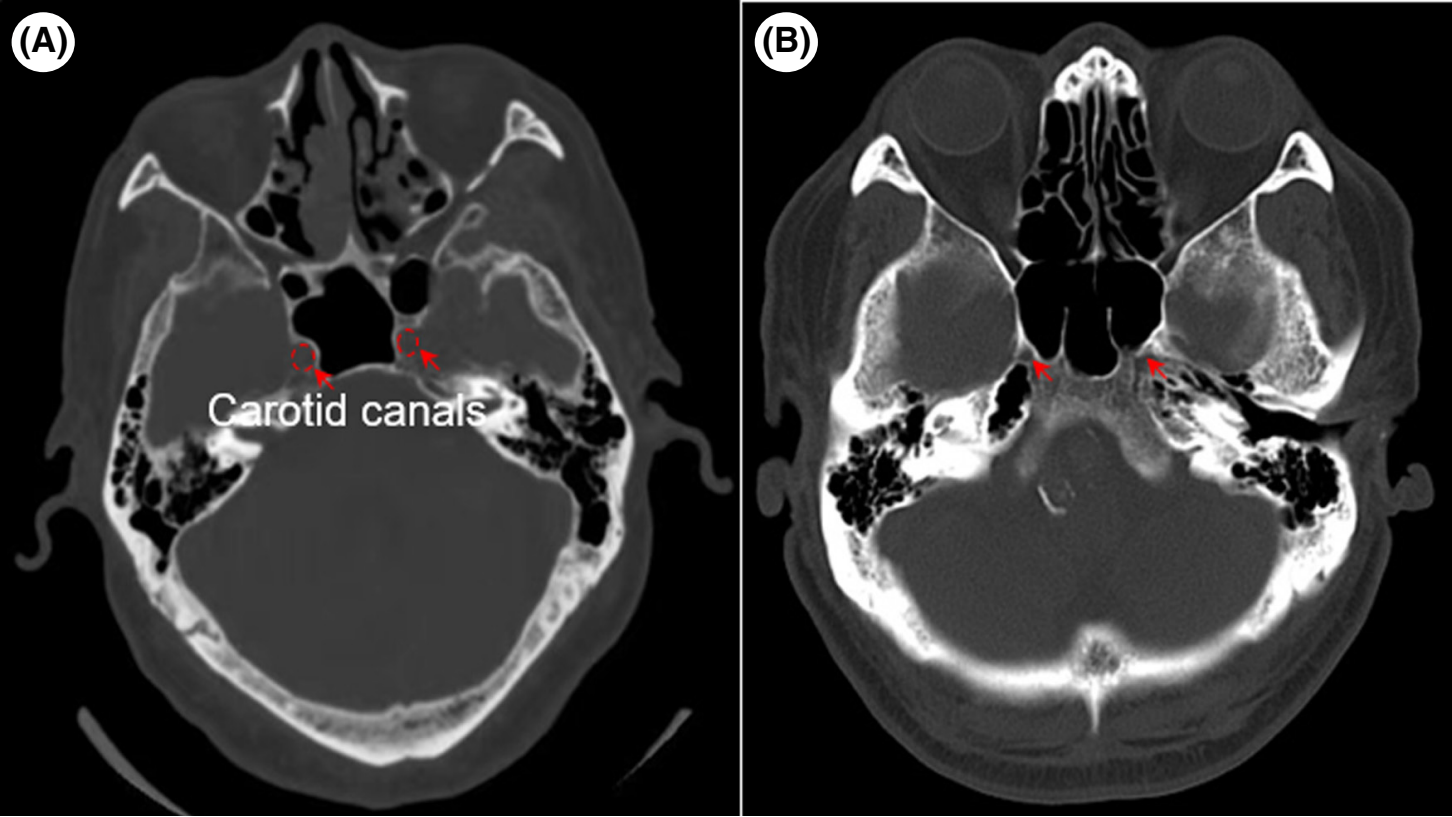
CT scan reveals hypoplastic carotid canals at both sides in the patient. (A) The ICA enters the skull through the carotid canals (red circle) in healthy control, (B) while the patient had none.

The absence of internal carotid artery (ICA) is a rare congenital vascular dysplasia with an incidence rate less than 0.01%, mostly shown by unilateral left.^1^ The patients with the absence of ICA generally show developmental delay, subarachnoid hemorrhage, headache, dizziness, eye disease, and tinnitus with compensatory vessel‐induced pulsive mass behind tympanum, basilar artery dilation, and abnormal ophthalmic artery originated from middle cerebral artery (MCA) or middle meningeal artery (MMA).^2^ Few patients with the absence of ICA are asymptomatic, in which the dilated vertebrobasilar artery and intact circle of Willis can be compensatory. The incidence of intracranial aneurysm associated with an increased hemodynamic load is 10‐folds higher than that of the normal population.^3^ The current treatment with the absence of ICA is symptomatic or rehabilitative, and collateral circulation reconstruction and surgical or endovascular repair to reduce the risk of aneurysm rupture are also important interventions to improve the survival rate of patients.^4^

In conclusion, we present here teaching neuroimages for the absence of bilateral ICA and multiple intracranial aneurysm in an old patient. The results also indicated that the vertebrobasilar artery and the intact circle of Willis play an important role in the compensation of global cerebral blood flow. However, changes in cerebral hemodynamics may increase the risk of aneurysmal genesis.

## AUTHOR CONTRIBUTIONS

**Yang Gao:** Data curation; funding acquisition; methodology; writing – original draft. **jianhua yuan:** Conceptualization; data curation; software; writing – original draft. **jianzhi wang:** Funding acquisition; project administration; writing – review and editing.

## FUNDING INFORMATION

Funded by the Wuhan Health Science Foundation (WX20Q04), the Fundamental Research Funds for the Central Universities (YCJJ202203019).

## CONFLICT OF INTEREST

The authors declare that there are no conflict of interests.

## CONSENT

Written informed consent was obtained from the patient to publish this report in accordance with the journal's patient consent policy.

## Data Availability

The data that support the findings of this study are available on request from the corresponding author. The data are not publicly available due to privacy or ethical restrictions.
